# *N*-(2-Methyl­phen­yl)benzene­sulfonamide

**DOI:** 10.1107/S1600536808024562

**Published:** 2008-08-06

**Authors:** B. Thimme Gowda, Sabine Foro, K. S. Babitha, Hartmut Fuess

**Affiliations:** a Department of Chemistry, Mangalore University, Mangalagangotri 574 199, Mangalore, India; b Institute of Materials Science, Darmstadt University of Technology, Petersenstrasse 23, D-64287 Darmstadt, Germany

## Abstract

In the title compound, C_13_H_13_NO_2_S, the conformation of the N—H bond is *anti* to the *ortho*-methyl group on the aniline ring, in contrast to the *syn* conformation observed with respect to the *ortho*-chloro group in *N*-(2-chloro­phen­yl)benzene­sulfonamide. The dihedral angle between the two benzene rings is 61.5 (1)°. Mol­ecules are linked into chains running along the *a* axis by N—H⋯O hydrogen bonds.

## Related literature

For related literature, see: Gelbrich *et al.*, 2007 ▶; Gowda *et al.* (2005 ▶, 2007*a* ▶,*b* ▶, 2008 ▶); Perlovich *et al.* (2006 ▶).
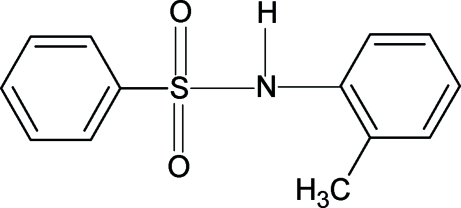


## Experimental

### 

#### Crystal data

C_13_H_13_NO_2_S
*M* *_r_* = 247.30
Orthorhombic, 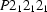

*a* = 6.4840 (6) Å
*b* = 8.6124 (8) Å
*c* = 21.915 (2) Å
*V* = 1223.8 (2) Å^3^
*Z* = 4
Mo *K*α radiation
μ = 0.25 mm^−1^
*T* = 299 (2) K
0.50 × 0.50 × 0.45 mm

#### Data collection

Oxford Diffraction Xcalibur diffractometer with Sapphire CCD detector
Absorption correction: multi-scan (*CrysAlis RED*; Oxford Diffraction, 2007 ▶) *T*_min_ = 0.884, *T*_max_ = 0.895
4256 measured reflections
2347 independent reflections
2164 reflections with *I* > 2σ(*I*)
*R*_int_ = 0.029

#### Refinement

*R*[*F* ^2^ > 2σ(*F* ^2^)] = 0.039
*wR*(*F* ^2^) = 0.106
*S* = 1.07
2347 reflections
184 parameters
H atoms treated by a mixture of independent and constrained refinement
Δρ_max_ = 0.22 e Å^−3^
Δρ_min_ = −0.35 e Å^−3^
Absolute structure: Flack (1983 ▶), 883 Friedel pairs
Flack parameter: 0.02 (10)

### 

Data collection: *CrysAlis CCD* (Oxford Diffraction, 2004 ▶); cell refinement: *CrysAlis RED* (Oxford Diffraction, 2007 ▶); data reduction: *CrysAlis RED*; program(s) used to solve structure: *SHELXS97* (Sheldrick, 2008 ▶); program(s) used to refine structure: *SHELXL97* (Sheldrick, 2008 ▶); molecular graphics: *PLATON* (Spek, 2003 ▶); software used to prepare material for publication: *SHELXL97*.

## Supplementary Material

Crystal structure: contains datablocks I, global. DOI: 10.1107/S1600536808024562/ci2650sup1.cif

Structure factors: contains datablocks I. DOI: 10.1107/S1600536808024562/ci2650Isup2.hkl

Additional supplementary materials:  crystallographic information; 3D view; checkCIF report

## Figures and Tables

**Table 1:** Hydrogen-bond geometry (Å, °)

*D*—H⋯*A*	*D*—H	H⋯*A*	*D*⋯*A*	*D*—H⋯*A*
N1—H1*N*⋯O1^i^	0.82 (3)	2.11 (3)	2.926 (3)	178 (3)

Symmetry code: (i) 
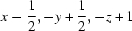
.

**Fig. 1.**
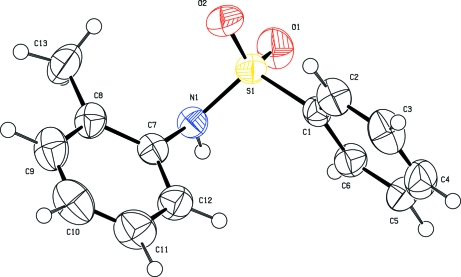
Molecular structure of the title compound, showing the atom labelling scheme. Displacement ellipsoids are drawn at the 50% probability level. H atoms are represented as small spheres of arbitrary radii.

**Fig. 2.**
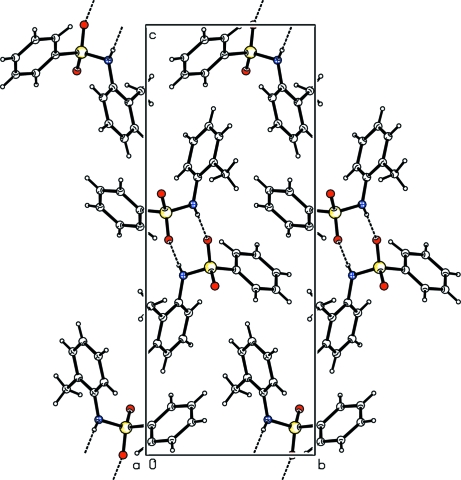
Part of the crystal structure of the title compound, viewed down the a axis. Hydrogen bonds are shown as dashed lines.

## supplementary crystallographic information

### Comment

As part of a study of the substituent effects on the crystal structures of *N*-(aryl)-sulfonamides the structure of *N*-(2-methylphenyl)-benzenesulfonamide (N2MPBSA) has been determined (Gowda *et al.*,  2007a,b, 2008). The conformations of the N—H and S═O bonds of the SO_2_—NH—C group are *trans* to each other (Fig. 1). Further, the conformation of the N—H bond is *anti* to the *ortho*-methyl group in the aniline benzene ring, in contrast to the *syn* conformation observed with respect to the *ortho*-chloro group in *N*-(2-chlorophenyl)-benzenesulfonamide (N2CPBSA) (Perlovich *et al.*, 2006). The two benzene rings are rotated relative to each other by 61.5 (1)° compared to the value of 49.1 (1)° in N2CPBSA. The other bond parameters in N2MPBSA are similar to those in N2CPBSA and other *N*-(aryl)-sulfonamides (Gelbrich *et al.*, 2007; Gowda *et al.*, 2007a,b, 2008).

In the crystal structure of N2MPBSA (Fig. 1), the molecules are linked into chains running along the *a* axis by N—H···O hydrogen bonds (Table 1).

### Experimental

A solution of benzene (10 ml) in chloroform (40 ml) was treated dropwise with chlorosulfonic acid (25 ml) at 273 K. After the initial evolution of hydrogen chloride subsided, the reaction mixture was brought to room temperature and poured into crushed ice in a beaker. The chloroform layer was separated, washed with cold water and allowed to evaporate slowly. The residual benzenesulfonylchloride was treated with *o*-toluidine in the stoichiometric ratio and boiled for 10 min. The reaction mixture was then cooled to room temperature and added to ice cold water (100 ml). The resultant solid *N*-(2-methylphenyl)-benzenesulfonamide was filtered under suction and washed thoroughly with cold water. It was then recrystallized to constant melting point from dilute ethanol. The purity of the compound was checked and characterized by recording its infrared and NMR spectra (Gowda *et al.*, 2005). Single crystals of the title compound used for X-ray diffraction studies were grown by slow evaporation of an ethanolic solution at room temperature.

### Refinement

H atoms of the methyl group were positioned geometrically and refined using a riding model, with C-H = 0.96 Å and U_iso_(H) = 1.5U_eq_(C). The remaining H atoms were located in a difference map and their positional parameters were refined [N-H = 0.82 (3) Å, C-H = 0.88 (4)–1.00 (4) Å] with U_iso_(H) = 1.2U_eq_(C,N). Three most deviating reflections (0 1 1, 0 1 2, 0 1 3) were omitted from the refinement.

### Crystal data

**Table:**

C_13_H_13_NO_2_S	*F*_000_ = 520
*M**_r_* = 247.30	*D*_x_ = 1.342 Mg m^−^^3^
Orthorhombic, *P*2_1_2_1_2_1_	Mo *K*α radiation λ = 0.71073 Å
Hall symbol: P 2ac 2ab	Cell parameters from 2747 reflections
*a* = 6.4840 (6) Å	θ = 2.4–28.0º
*b* = 8.6124 (8) Å	µ = 0.25 mm^−^^1^
*c* = 21.915 (2) Å	*T* = 299 (2) K
*V* = 1223.8 (2) Å^3^	Prism, colourless
*Z* = 4	0.50 × 0.50 × 0.45 mm

### Data collection

**Table:**

Oxford Diffraction Xcalibur diffractometer with Sapphire CCD detector	2347 independent reflections
Radiation source: fine-focus sealed tube	2164 reflections with *I* > 2σ(*I*)
Monochromator: graphite	*R*_int_ = 0.029
*T* = 299(2) K	θ_max_ = 26.4º
ω and φ scans	θ_min_ = 3.3º
Absorption correction: multi-scan(CrysAlis RED; Oxford Diffraction, 2007)	*h* = −7→5
*T*_min_ = 0.884, *T*_max_ = 0.895	*k* = −10→9
4256 measured reflections	*l* = −18→27

### Refinement

**Table:**

Refinement on *F*^2^	Hydrogen site location: inferred from neighbouring sites
Least-squares matrix: full	H atoms treated by a mixture of independent and constrained refinement
*R*[*F*^2^ > 2σ(*F*^2^)] = 0.039	*w* = 1/[σ^2^(*F*_o_^2^) + (0.06*P*)^2^ + 0.3217*P*] where *P* = (*F*_o_^2^ + 2*F*_c_^2^)/3
*wR*(*F*^2^) = 0.106	(Δ/σ)_max_ = 0.014
*S* = 1.08	Δρ_max_ = 0.22 e Å^−^^3^
2347 reflections	Δρ_min_ = −0.35 e Å^−^^3^
184 parameters	Extinction correction: none
Primary atom site location: structure-invariant direct methods	Absolute structure: Flack (1983), 883 Friedel pairs
Secondary atom site location: difference Fourier map	Flack parameter: 0.02 (10)

### Special details

**Table:**

Geometry. All e.s.d.'s (except the e.s.d. in the dihedral angle between two l.s. planes) are estimated using the full covariance matrix. The cell e.s.d.'s are taken into account individually in the estimation of e.s.d.'s in distances, angles and torsion angles; correlations between e.s.d.'s in cell parameters are only used when they are defined by crystal symmetry. An approximate (isotropic) treatment of cell e.s.d.'s is used for estimating e.s.d.'s involving l.s. planes.
Refinement. Refinement of *F*^2^ against ALL reflections. The weighted *R*-factor *wR* and goodness of fit *S* are based on *F*^2^, conventional *R*-factors *R* are based on *F*, with *F* set to zero for negative *F*^2^. The threshold expression of *F*^2^ > σ(*F*^2^) is used only for calculating *R*-factors(gt) *etc*. and is not relevant to the choice of reflections for refinement. *R*-factors based on *F*^2^ are statistically about twice as large as those based on *F*, and *R*- factors based on ALL data will be even larger.

### Fractional atomic coordinates and isotropic or equivalent isotropic displacement parameters (Å^2^)

**Table:**

	*x*	*y*	*z*	*U*_iso_*/*U*_eq_	
S1	0.47917 (9)	0.38178 (7)	0.43612 (2)	0.04060 (17)	
O1	0.5270 (3)	0.3651 (2)	0.49979 (8)	0.0608 (5)	
O2	0.6400 (3)	0.4082 (2)	0.39280 (9)	0.0589 (5)	
N1	0.3623 (3)	0.2218 (2)	0.41762 (8)	0.0391 (4)	
H1N	0.271 (5)	0.195 (3)	0.4409 (13)	0.047*	
C1	0.2996 (3)	0.5346 (2)	0.43031 (10)	0.0362 (5)	
C2	0.3264 (5)	0.6510 (3)	0.38754 (11)	0.0493 (6)	
H2	0.436 (5)	0.649 (3)	0.3609 (13)	0.059*	
C3	0.1819 (6)	0.7692 (3)	0.38473 (14)	0.0617 (8)	
H3	0.199 (5)	0.847 (4)	0.3560 (15)	0.074*	
C4	0.0157 (5)	0.7710 (3)	0.42368 (14)	0.0600 (7)	
H4	−0.075 (5)	0.847 (4)	0.4198 (14)	0.072*	
C5	−0.0106 (4)	0.6534 (3)	0.46522 (13)	0.0569 (7)	
H5	−0.132 (5)	0.657 (4)	0.4885 (14)	0.068*	
C6	0.1308 (4)	0.5347 (3)	0.46890 (11)	0.0462 (6)	
H6	0.111 (5)	0.447 (3)	0.4956 (12)	0.055*	
C7	0.3086 (4)	0.1940 (3)	0.35437 (10)	0.0378 (5)	
C8	0.4396 (4)	0.1005 (3)	0.31992 (10)	0.0455 (5)	
C9	0.3803 (6)	0.0680 (4)	0.25957 (12)	0.0601 (7)	
H9	0.469 (5)	0.004 (4)	0.2348 (15)	0.072*	
C10	0.2019 (6)	0.1273 (4)	0.23580 (13)	0.0689 (9)	
H10	0.181 (5)	0.100 (4)	0.1956 (16)	0.083*	
C11	0.0750 (6)	0.2190 (4)	0.27027 (14)	0.0668 (9)	
H11	−0.059 (6)	0.265 (4)	0.2564 (17)	0.080*	
C12	0.1286 (4)	0.2529 (3)	0.33042 (12)	0.0522 (6)	
H12	0.043 (5)	0.318 (4)	0.3561 (14)	0.063*	
C13	0.6314 (5)	0.0302 (4)	0.34651 (15)	0.0661 (8)	
H13A	0.5953	−0.0343	0.3806	0.079*	
H13B	0.7222	0.1114	0.3599	0.079*	
H13C	0.6994	−0.0314	0.3160	0.079*	

### Atomic displacement parameters (Å^2^)

**Table:**

	*U*^11^	*U*^22^	*U*^33^	*U*^12^	*U*^13^	*U*^23^
S1	0.0369 (3)	0.0429 (3)	0.0419 (3)	0.0025 (2)	−0.0041 (2)	−0.0025 (2)
O1	0.0723 (13)	0.0611 (11)	0.0490 (9)	0.0122 (11)	−0.0249 (9)	−0.0039 (8)
O2	0.0390 (9)	0.0623 (12)	0.0755 (12)	−0.0041 (9)	0.0128 (9)	−0.0048 (9)
N1	0.0410 (10)	0.0420 (10)	0.0342 (9)	−0.0013 (9)	0.0041 (8)	−0.0002 (7)
C1	0.0388 (11)	0.0359 (11)	0.0338 (10)	0.0002 (9)	−0.0040 (9)	−0.0036 (9)
C2	0.0624 (16)	0.0477 (14)	0.0377 (11)	−0.0049 (12)	0.0043 (11)	0.0037 (10)
C3	0.090 (2)	0.0435 (15)	0.0517 (15)	0.0008 (15)	−0.0140 (15)	0.0098 (12)
C4	0.0627 (17)	0.0513 (14)	0.0661 (16)	0.0170 (14)	−0.0170 (15)	−0.0092 (12)
C5	0.0451 (15)	0.0594 (16)	0.0663 (16)	0.0085 (13)	0.0036 (13)	−0.0143 (12)
C6	0.0468 (14)	0.0462 (14)	0.0454 (12)	−0.0019 (12)	0.0068 (11)	−0.0003 (10)
C7	0.0427 (12)	0.0373 (11)	0.0333 (10)	−0.0047 (9)	0.0036 (9)	0.0033 (9)
C8	0.0520 (13)	0.0447 (12)	0.0398 (11)	0.0000 (11)	0.0103 (9)	0.0012 (10)
C9	0.081 (2)	0.0590 (16)	0.0408 (12)	−0.0032 (15)	0.0152 (14)	−0.0044 (12)
C10	0.095 (2)	0.075 (2)	0.0368 (12)	−0.0140 (19)	−0.0078 (15)	−0.0007 (14)
C11	0.068 (2)	0.077 (2)	0.0549 (16)	0.0008 (16)	−0.0181 (15)	0.0022 (15)
C12	0.0490 (15)	0.0592 (15)	0.0484 (13)	0.0050 (14)	−0.0045 (12)	−0.0036 (11)
C13	0.0553 (17)	0.0716 (19)	0.0713 (18)	0.0198 (16)	0.0099 (15)	−0.0114 (15)

### Geometric parameters (Å, °)

**Table:**

S1—O2	1.4284 (19)	C6—H6	0.96 (3)
S1—O1	1.4365 (17)	C7—C12	1.376 (4)
S1—N1	1.624 (2)	C7—C8	1.393 (3)
S1—C1	1.762 (2)	C8—C9	1.405 (4)
N1—C7	1.449 (3)	C8—C13	1.501 (4)
N1—H1N	0.82 (3)	C9—C10	1.368 (5)
C1—C6	1.383 (3)	C9—H9	0.97 (3)
C1—C2	1.384 (3)	C10—C11	1.368 (5)
C2—C3	1.385 (4)	C10—H10	0.92 (3)
C2—H2	0.92 (3)	C11—C12	1.394 (4)
C3—C4	1.375 (5)	C11—H11	1.00 (4)
C3—H3	0.93 (3)	C12—H12	0.97 (3)
C4—C5	1.373 (4)	C13—H13A	0.96
C4—H4	0.88 (4)	C13—H13B	0.96
C5—C6	1.376 (4)	C13—H13C	0.96
C5—H5	0.94 (3)		
			
O2—S1—O1	120.26 (13)	C1—C6—H6	118.4 (18)
O2—S1—N1	108.05 (11)	C12—C7—C8	121.6 (2)
O1—S1—N1	104.97 (11)	C12—C7—N1	120.5 (2)
O2—S1—C1	108.38 (11)	C8—C7—N1	117.8 (2)
O1—S1—C1	106.73 (11)	C7—C8—C9	117.3 (2)
N1—S1—C1	107.90 (10)	C7—C8—C13	121.9 (2)
C7—N1—S1	119.41 (15)	C9—C8—C13	120.8 (3)
C7—N1—H1N	112 (2)	C10—C9—C8	121.0 (3)
S1—N1—H1N	115 (2)	C10—C9—H9	120.4 (19)
C6—C1—C2	120.9 (2)	C8—C9—H9	118.6 (19)
C6—C1—S1	118.63 (17)	C11—C10—C9	121.0 (3)
C2—C1—S1	120.5 (2)	C11—C10—H10	126 (2)
C1—C2—C3	118.5 (3)	C9—C10—H10	113 (2)
C1—C2—H2	120.8 (19)	C10—C11—C12	119.5 (3)
C3—C2—H2	120.7 (19)	C10—C11—H11	126 (2)
C4—C3—C2	120.7 (3)	C12—C11—H11	115 (2)
C4—C3—H3	120 (2)	C7—C12—C11	119.7 (3)
C2—C3—H3	119 (2)	C7—C12—H12	118.6 (18)
C5—C4—C3	120.0 (3)	C11—C12—H12	121.8 (18)
C5—C4—H4	122 (2)	C8—C13—H13A	109.5
C3—C4—H4	118 (2)	C8—C13—H13B	109.5
C4—C5—C6	120.3 (3)	H13A—C13—H13B	109.5
C4—C5—H5	116 (2)	C8—C13—H13C	109.5
C6—C5—H5	123 (2)	H13A—C13—H13C	109.5
C5—C6—C1	119.5 (2)	H13B—C13—H13C	109.5
C5—C6—H6	122.0 (18)		
			
O2—S1—N1—C7	−45.0 (2)	C2—C1—C6—C5	−0.9 (4)
O1—S1—N1—C7	−174.43 (18)	S1—C1—C6—C5	179.7 (2)
C1—S1—N1—C7	72.0 (2)	S1—N1—C7—C12	−84.6 (3)
O2—S1—C1—C6	−179.00 (18)	S1—N1—C7—C8	98.4 (2)
O1—S1—C1—C6	−48.1 (2)	C12—C7—C8—C9	−0.2 (4)
N1—S1—C1—C6	64.23 (19)	N1—C7—C8—C9	176.8 (2)
O2—S1—C1—C2	1.6 (2)	C12—C7—C8—C13	−177.3 (3)
O1—S1—C1—C2	132.5 (2)	N1—C7—C8—C13	−0.3 (3)
N1—S1—C1—C2	−115.2 (2)	C7—C8—C9—C10	0.6 (4)
C6—C1—C2—C3	0.9 (4)	C13—C8—C9—C10	177.7 (3)
S1—C1—C2—C3	−179.7 (2)	C8—C9—C10—C11	−0.6 (5)
C1—C2—C3—C4	0.2 (4)	C9—C10—C11—C12	0.3 (5)
C2—C3—C4—C5	−1.2 (4)	C8—C7—C12—C11	−0.1 (4)
C3—C4—C5—C6	1.2 (4)	N1—C7—C12—C11	−177.1 (3)
C4—C5—C6—C1	−0.1 (4)	C10—C11—C12—C7	0.1 (5)

### Hydrogen-bond geometry (Å, °)

**Table:**

*D*—H···*A*	*D*—H	H···*A*	*D*···*A*	*D*—H···*A*
N1—H1N···O1^i^	0.82 (3)	2.11 (3)	2.926 (3)	178 (3)

Symmetry codes: (i) *x*−1/2, −*y*+1/2, −*z*+1.
